# Supplementary material for: Role of Secreted Frizzled-Related Protein 1 in Early Breast Carcinogenesis and Breast Cancer Aggressiveness
Source: Cancers (Basel). 2023 Apr 12;15(8):2251. doi: 10.3390/cancers15082251 (PMC10136791; doi:10.3390/cancers15082251)
Supplement: Supplementary file 1 [file cancers-15-02251-s001.zip › cancers-2294428-supplementary.pdf]

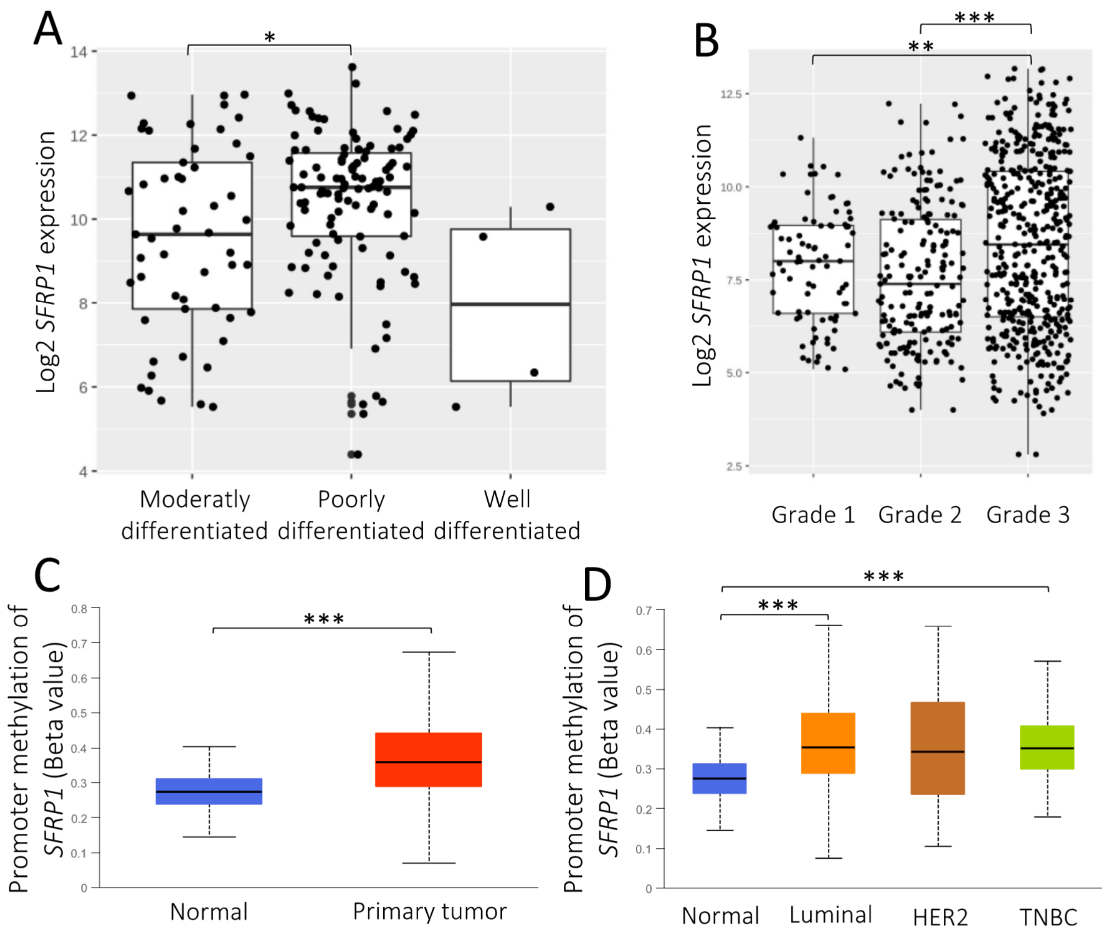

Figure S1. *SFRP1* expression pattern across breast cancer tissues and its promoter methylation status. (A) *SFRP1* expression profile in breast tumoral tissue in poorly differentiated tumors compared to moderately (log2 FC = 0.90; p-value < 0.05) and well differentiated (log2 FC = 2.49; p-value = 0.12) tumors. (B) *SFRP1* expression pattern regarding breast tumor grading. Figures 1A-B were obtained from http://gent2.appex.kr/gent2/. (C) *SFRP1* promoter methylation profile in normal (n = 97) and tumoral (n = 793) breast tissue from the TCGA database. (D) *SFRP1* promoter methylation profile in normal breast (n = 97), luminal (n = 393), HER2 (n = 17) and triple negative (n = 84) breast cancer. Breast cancer molecular subtypes were determined by immunohistochemistry. Figures C and D were drawn with the ULCAN online tool (http://ualcan.path.uab.edu/).

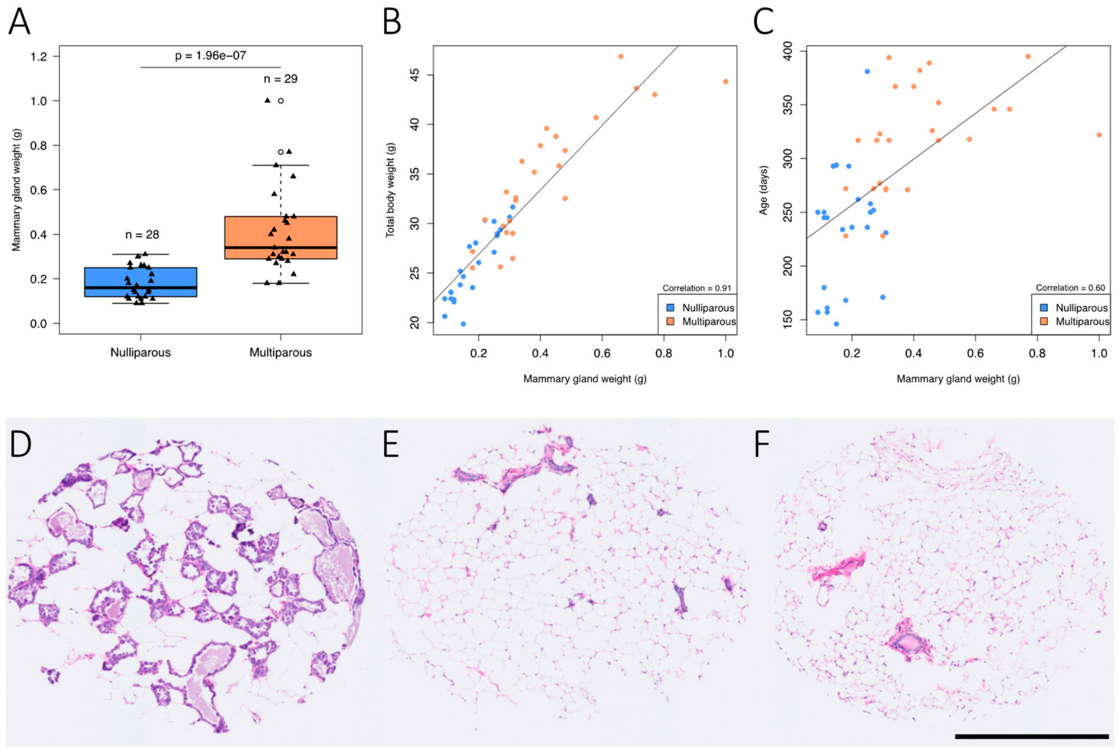

Figure S2. Characterization of mammary gland obtained from nulliparous and multiparous mice. (A) Mammary glands weight (g) according to parity status (n = 57). (B) Correlation between mice total body weight (g) and mammary glands weight (g) according to parity status (n = 57). (C) Correlation between mice age (days) and mammary glands weight (g) according to parity status (n = 57). H&E staining of mammary glands obtained from multiparous mice (D) 48h post weaning, and (E) 25 days post-weaning. (F) H&E staining of mammary glands obtained from a nulliparous mouse. Scale bar = 500 µm

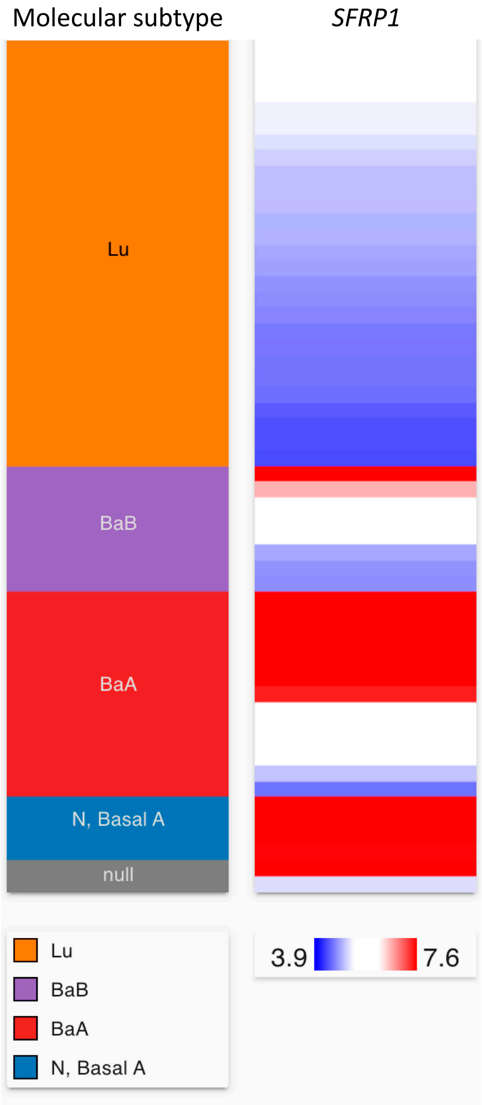

Figure S3. *SFRP1* expression pattern across breast cancer cell lines according to their molecular subtype. Lu = luminal, BaB = basal A, BaB = basal B, N, Basal A = normal. The figure was drawn with UCSC Xena online tool (https://xenabrowser.net/).

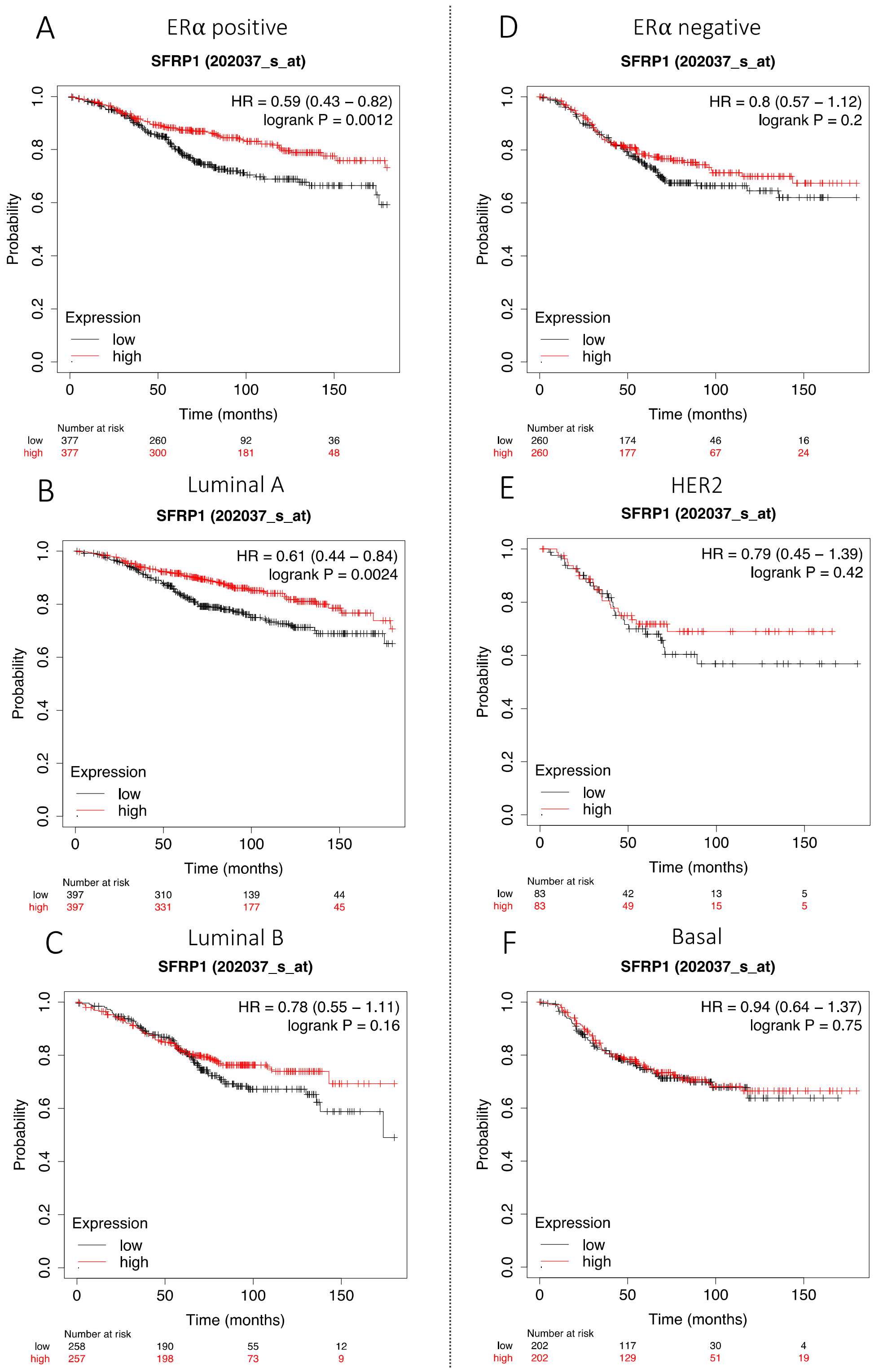

Figure S4. Association between *SFRP1* expression and breast cancer outcomes. Association between *SFRP1* expression and ERα positive (A); ERα negative (B); luminal A (C); luminal B (D); HER2 (E); basal (F) breast cancer molecular subtypes and breast cancer overall survival. Kaplan-Meier curves were drawn online with **https://kmplot.com/analysis/**.

Table S1: Primers sequences, targets, and amplicons size.

Gene Symbol	Description	Size (bp)	Primer sequence 5'→3' S/AS
SFRP1	*Secreted frizzled -related protein 1 (Homo sapiens)*	111	TCTACCCGTGTCGCTGGCTCT/ CCTCGGGGAACTTGTCACACTTAA
ESR1	*Estrogen receptor 1 (Homo sapiens)*	71	TCTTGGACAGGAACCAGGA/ TGATGTAGCCAGCAGCATGT
ESR2	*Estrogen receptor 2 (Homo sapiens)*		
STAT3	*Signal transducer and activator of transcription 3 (Homo sapiens)*	133	CCTTTGAGACCGAGGTGTATCACC / GGTCAGCATGTTGTACCACAGG
c-MYC	*MYC Proto-Oncogene (Homo sapiens)*	122	GTCCTCGGATTCTCTGCTCTC/ CATCTTCTTGTTCCTCCTCAGAGT
c-JUN	*JUN Proto-Oncogene (Homo sapiens)*	126	CCAACTCATGCTAACGCAGC/ TCTCTCCGTCGCAACTTGTC
ATP5O	*ATP synthase, H+ transporting, mitochondrial F1 complex, O subunit (Homo sapiens)*	103	ATTGAAGGTCGCTATGCCACAG/ CCTTCAGGATTTGTGCTACTCTCA
HPRT1	*Hypoxanthine phosphoribosyltransferase 1 (Homo sapiens)*	86	CGTCGTGATTAGTGATGATGAACCA/ ACACCCTTTCCAAATCCTCAGC
GAPDH	*Glyceraldehyde-3-phosphate dehydrogenase (Homo sapiens)*	93	CGGGGCTCTCCAGAACATCAT/ ATGCCAGTGAGCTTCCCGTTC
Sfrp1	*Secreted frizzled -related protein 1 (Mus musculus)*	108	CTGGCCCGAGATGCTCAAAT/ CACACGGTTGTACCTTGGGG
Esr1	*Estrogen receptor 1 (Mus musculus)*	97	AAGAGAGTGCCAGGCTTTGG/ GCCAGACGAGACCAATCATCA
Esr2	*Estrogen receptor 2 (Mus musculus)*	111	GAGAATGTCCACCCGCTAGG/ TGTCTTGCGTAGGTCTCAGC
Stat3	*Signal transducer and activator of transcription 3 (Mus musculus)*	115	TGGACCGTCTGGAAAACTGG/ ATAGGGTCGCCCTTGTAGGA
c-Myc	*Myc Proto-Oncogene (Mus musculus)*	130	CCTTCTCTCCTTCCTCGGAC/ TCATCTTCTTGCTCTTCTTCAGAGT
c-Jun	*Jun Proto-Oncogene (Mus musculus)*	119	TGGGCACATCACCACTACAC/ TCTGGCTATGCAGTTCAGCC
Pum1	*Pumilio RNA binding family member 1 (Mus musculus)*	267	AACATCGATGGCCTACAGGG/ GACCAGGTCTTCTCTGCACC
Tbp1	*TATA binding protein 1 (Mus musculus)*	94	GTTGGGCTTCCCAGCTAAGT/ CACAAGGCCTTCCAGCCTTA
